# The Impact of Diet on Parkinson's Disease: A Systematic Review

**DOI:** 10.7759/cureus.70337

**Published:** 2024-09-27

**Authors:** Luqman Anwar, Ejaz Ahmad, Muhammad Imtiaz, Muhammad Ahmad, Muhammad Faisal Aziz, Talha ibad

**Affiliations:** 1 Neurology, Mayo Hospital, Lahore, PAK; 2 Neurology, Gangaram Hospital, Lahore, PAK; 3 Medicine, Punjab Medical College, Faisalabad, PAK; 4 Internal Medicine, District Headquarter (DHQ) Hospital Layyah, Layyah, PAK; 5 Medicine, Services Hospital Lahore, Lahore, PAK

**Keywords:** antioxidants, diet, dietary patterns, gut microbiome, neuroprotection, nutrition, parkinson's disease

## Abstract

Parkinson's disease (PD) is a progressive neurodegenerative disorder with complex etiology. Emerging evidence suggests that diet may play a role in PD risk, progression, and symptom management. However, the relationship between dietary factors and PD remains poorly understood. This systematic review aimed to synthesize and evaluate the current evidence on the associations between dietary patterns, specific nutrients, and PD risk, progression, and symptom management. We conducted a comprehensive literature search in major databases for studies published up to 2024. Eligible studies included prospective cohorts, case-control studies, randomized controlled trials, and cross-sectional analyses investigating the relationship between diet and PD. Data extraction and quality assessment were performed independently by two reviewers. Eleven studies met the inclusion criteria. Adherence to healthy dietary patterns, particularly those rich in fruits, vegetables, whole grains, and fish, was consistently associated with reduced PD risk. Conversely, Western-style diets high in processed foods and red meat were linked to increased risk. Specific nutrients, including antioxidants and vitamins K and C, showed potential neuroprotective effects, while high iron intake was associated with increased PD risk. Diet quality was found to influence PD symptoms, particularly non-motor symptoms like constipation. Emerging evidence suggested a role for the gut microbiome in mediating diet-PD relationships. Specialized diets, such as ketogenic and low-carbohydrate diets, showed promise in managing PD symptoms in small-scale studies. This review provides evidence for the significant role of diet in PD risk, progression, and symptom management. Dietary interventions have the potential to serve as complementary approaches to existing PD therapies. However, the complex nature of the diet-PD relationship necessitates further research, particularly well-designed long-term randomized controlled trials, to develop evidence-based, personalized dietary recommendations for PD prevention and management.

## Introduction and background

Parkinson's disease (PD) is a progressive neurodegenerative disorder that affects 6.1 million people worldwide in 2016 with mortality of 3.2 million, with an estimated prevalence of 1%-2% in individuals over 65 years of age [1]. Characterized by motor symptoms such as tremor, rigidity, bradykinesia, and postural instability, PD also presents a wide array of non-motor symptoms, including cognitive impairment, depression, and gastrointestinal disturbances [2]. The etiology of PD is complex and multifactorial, involving a combination of genetic susceptibility and environmental factors [3,4].

In recent years, there has been growing interest in the role of diet as a modifiable risk factor and potential therapeutic target in PD [5]. This interest stems from several lines of evidence suggesting that dietary factors may influence the development, progression, and symptom management of PD through various mechanisms, including oxidative stress modulation, neuroinflammation, and alterations in the gut microbiome [6]. Oxidative stress has long been implicated in the pathogenesis of PD, with the loss of dopaminergic neurons in the substantia nigra pars compacta being particularly vulnerable to oxidative damage [7]. Dietary antioxidants, such as vitamins C and E, carotenoids, and polyphenols, have been hypothesized to provide neuroprotection by scavenging free radicals and reducing oxidative stress [8]. Several epidemiological studies have suggested an inverse association between the intake of antioxidant-rich foods and PD risk [9].

The gut-brain axis has emerged as a crucial pathway in PD pathophysiology, with mounting evidence indicating that gastrointestinal dysfunction and alterations in the gut microbiome may precede motor symptoms by years or even decades [10,11]. Dietary factors can significantly influence the composition and function of the gut microbiome, potentially modulating PD risk and progression [12]. For instance, high-fiber diets have been associated with increased production of short-chain fatty acids, which may have neuroprotective properties [13].

Dietary patterns, rather than individual nutrients, have gained attention in PD research due to their potential to capture the complexity of nutrient interactions and overall dietary quality [14]. The Mediterranean diet, characterized by high consumption of fruits, vegetables, whole grains, legumes, nuts, and olive oil, has been associated with reduced risk of several neurodegenerative diseases, including PD [15]. Similarly, plant-based diets and those rich in polyunsaturated fatty acids have shown promise in epidemiological studies [16,17]. Specific nutrients have also been investigated for their potential neuroprotective or detrimental effects in PD. For example, caffeine consumption has been consistently associated with a lower risk of PD in numerous epidemiological studies [18]. Conversely, high intake of dairy products has been linked to an increased risk of PD in some studies, although the mechanisms underlying this association remain unclear [19].

The potential role of dietary factors in managing PD symptoms and improving quality of life has also garnered attention. Protein redistribution diets, which aim to optimize levodopa absorption by manipulating protein intake, have shown some efficacy in managing motor fluctuations in PD patients [20]. Additionally, dietary interventions targeting specific non-motor symptoms, such as constipation and cognitive impairment, have been explored with varying degrees of success [21-23]. Despite the growing body of research, the relationship between diet and PD remains complex and, in many aspects, inconclusive. Methodological challenges, such as the difficulty in accurately assessing long-term dietary habits and the potential for reverse causation in observational studies, have contributed to inconsistent findings across studies [24,25]. Moreover, the heterogeneity of PD itself, with varying clinical presentations and rates of progression, adds another layer of complexity to understanding the impact of diet on the disease [26]. The interaction between diet and genetics in PD risk and progression is an emerging area of research. Nutrigenomics studies have begun to explore how dietary factors may modulate gene expression and interact with genetic risk factors for PD [27]. This line of inquiry holds promise for developing personalized nutritional strategies for PD prevention and management.

Given the potential of dietary interventions as a low-risk, cost-effective approach to PD prevention and management, there is a critical need for a comprehensive synthesis of the current evidence. This review aims to evaluate the impact of diet on various aspects of PD, including disease risk, progression, symptom management, and quality of life. By examining a wide range of dietary factors, from specific nutrients to overall dietary pattern, and their effects on multiple PD-related outcomes, this review seeks to provide a nuanced understanding of the diet-PD relationship. Furthermore, this review will explore the potential mechanisms underlying the observed associations between diet and PD, with a particular focus on oxidative stress, neuroinflammation, gut microbiome alterations, and mitochondrial dysfunction [28,29]. By elucidating these mechanisms, we aim to identify promising avenues for future research and potential targets for dietary interventions. The findings of this review have important implications for clinical practice, public health strategies, and future research directions. As the global burden of PD continues to grow with an aging population [30], identifying modifiable risk factors and effective management strategies is of paramount importance. Dietary interventions, if proven effective, could offer a complementary approach to existing pharmacological and surgical treatments for PD, potentially improving patient outcomes and quality of life [31].

In summary, this review aims to provide a comprehensive analysis of the current evidence on the impact of diet on PD. By synthesizing data from a wide range of studies and examining multiple aspects of the diet-PD relationship, we hope to offer valuable insights for researchers, clinicians, and individuals affected by PD, ultimately contributing to the development of evidence-based dietary recommendations for PD prevention and management.

Aim and objectives

The aim of this study is to comprehensively evaluate the relationship between dietary factors and PD, including its risk, progression, and symptom management. The objectives are to assess the impact of various dietary patterns on PD risk and progression and to evaluate the role of specific nutrients, particularly antioxidants and micronutrients, in PD prevention and management. Furthermore, this study aims to investigate the relationship between diet quality and PD symptoms and to explore the potential role of the gut microbiome as a mediator between diet and PD. Moreover, this study intend to examine the effects of specialized diets (e.g., Mediterranean, ketogenic) on PD outcomes, assess the impact of dietary factors on cognitive function and nutritional status in PD patients, and identify potential mechanisms linking nutrition and PD pathogenesis.

## Review

Research question and protocol

Research Question (PICO)

Population: Adults (aged 18 and above), including those at risk for PD and those diagnosed with PD.

Intervention/Exposure: Dietary factors (patterns, specific nutrients, diet quality, specialized diets).

Comparison: Varied based on study design (e.g., different levels of dietary factor exposure, healthy controls).

Outcomes: PD risk, disease progression, symptom severity (motor and non-motor), quality of life, gut microbiome composition, cognitive function. The description of PICO components is aligned in table 1 below.

**Table 1:** PICO framework PICO: Patient, intervention, comparison, outcome; PD: Parkinson's disease

PICO element	Description
Population	Adults (≥18 years), including those at risk for PD and diagnosed PD patients
Intervention/exposure	Dietary patterns, specific nutrients (e.g., antioxidants, micronutrients), diet quality, specialized diets (e.g., Mediterranean, ketogenic)
Comparison	Varied by study: different levels of dietary exposure, healthy controls, standard diet
Outcomes	Primary: PD risk, disease progression; secondary: symptom severity (motor and non-motor), quality of life, gut microbiome composition, cognitive function

Protocol

This review was conducted following the Preferred Reporting Items for Systematic Reviews and Meta-Analyses (PRISMA) guidelines.

Selection Criteria

The study inclusion criteria encompassed a range of research designs, including randomized controlled trials, cohort studies, case-control studies, and cross-sectional studies. The focus was on adult populations, specifically individuals aged 18 years and older. This broad age range allowed for a comprehensive examination of dietary factors across different life stages and their potential impact on PD. The exposure of interest was any dietary factor, which provided a wide scope for analysis. This included dietary patterns, specific nutrients, overall diet quality, and specialized diets. By considering such a diverse range of dietary exposures, the review aimed to capture the multifaceted nature of nutritional influences on PD.

The outcomes examined in the review were equally comprehensive, encompassing PD risk, disease progression, symptoms, quality of life, gut microbiome composition, and cognitive function. This broad spectrum of outcomes allowed for a holistic understanding of how diet may influence various aspects of PD, from initial risk to long-term management and quality of life. To ensure accessibility and understanding, the review was limited to studies published in English. However, there was no restriction on publication date, allowing for the inclusion of both seminal works and the most recent research in the field. Several exclusion criteria were applied to maintain the focus and relevance of the review. Animal studies were excluded to ensure direct applicability to human populations. Finally, conference abstracts, reviews, unpublished studies, and grey literature were excluded to maintain a high standard of peer-reviewed evidence.

These criteria were designed to create a comprehensive yet focused review of the relationship between diet and PD, providing a solid foundation for future research and potential dietary interventions in PD prevention and management.

Search Strategy

The search strategy utilized multiple databases: PubMed, Embase, Cochrane Library, and Web of Science. MeSH terms and keywords were combined in the search string: (Parkinson's disease OR Parkinsonism) AND (diet OR nutrition OR food OR dietary pattern OR antioxidants OR micronutrients OR gut microbiome). Additional sources included reference lists of included studies and relevant reviews.

The review considers a broad spectrum of outcomes related to PD, including risk, progression, symptoms, quality of life, gut microbiome changes, and cognitive function. The MeSH terms cover these various aspects, from general concepts like "Risk" to more specific outcomes like "Cognition." PICO along with the corresponding MeSH terms are given in Table 2.

**Table 2:** PICO table with descriptions and corresponding MeSH terms PICO: Patient, intervention, comparison, outcome; PD: Parkinson's disease

PICO element	Description	MeSH terms
Population (P)	Adults aged 18 years and older, including those with PD, at risk of PD, or healthy individuals	"Adult" (Mesh), "Aged" (Mesh), "Parkinson Disease" (Mesh)
Intervention/exposure (I)	Any dietary factor, including dietary patterns, specific nutrients, diet quality, and specialized diets	"Diet" (Mesh), "Food" (Mesh), "Nutritional Status" (Mesh), "Dietary Supplements" (Mesh), "Antioxidants" (Mesh), "Micronutrients" (Mesh), "Gastrointestinal Microbiome" (Mesh)
Comparison (C)	Varies by study design. May include comparison to different dietary patterns, levels of nutrient intake, or non-exposure groups	Not applicable (varies by study)
Outcomes (O)	PD risk, disease progression, symptoms, quality of life, gut microbiome composition, cognitive function	"Risk" (Mesh), "Disease Progression" (Mesh), "Signs and Symptoms" (Mesh), "Quality of Life" (Mesh), "Gastrointestinal Microbiome" (Mesh), "Cognition" (Mesh)

Study Selection

**Figure 1:**
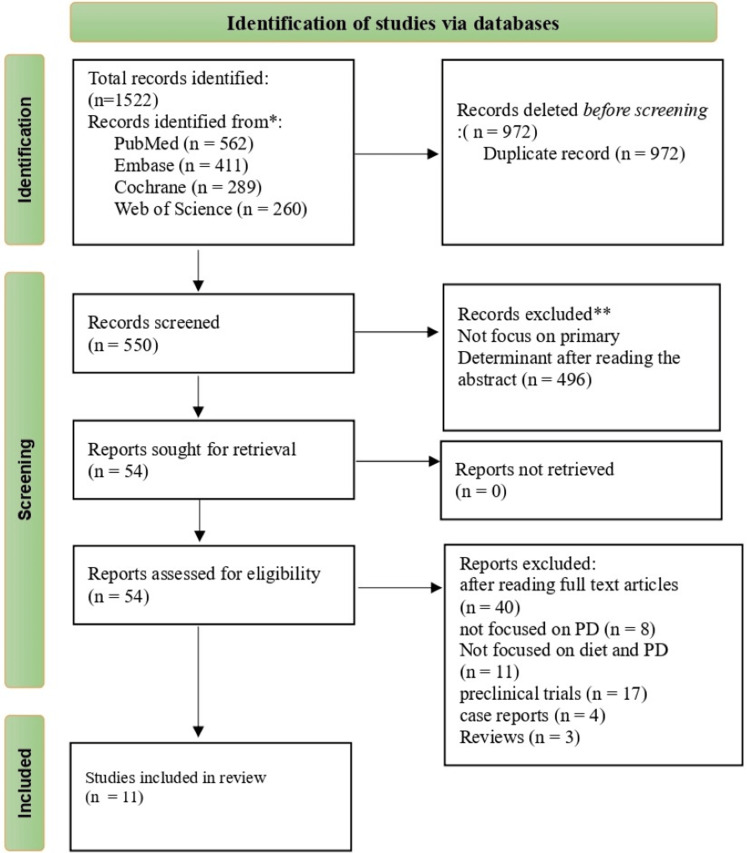
PRISMA chart PRISMA: Preferred Reporting Items for Systematic Reviews and Meta-Analyses

Two independent reviewers screened titles and abstracts following the PRISMA guidelines (Figure 1) [32]. Any disagreement in this process was resolved through a discussion or a third reviewer consultation.

Data Extraction

Data was extracted by two independent reviewers, and any disagreement was mutually discussed and resolved. A third reviewer was only involved, in case the controversy between two primary reviewers was not resolved by their mutual discussion. A predesigned, piloted form was used to extract data including the study characteristics, population details, exposure/intervention specifics, outcome measures, and key findings.

Outcome Measures

The primary outcome measures were the PD risk (incidence or prevalence) and disease progression. While the secondary outcomes measures were symptom severity (motor and non-motor), quality of life (e.g., PDQ-39), gut microbiome composition, and cognitive function.

Quality Assessment

This review included various types of study design. Thus, the Mixed Method Appraisal Tool (MMAT) was selected, as it can assess the quality of various study designs including prospective cohorts, case-control studies, cross-sectional analyses, and reviews [33]. This tool reports the quality of study in percentage; higher percentage indicates a higher quality study and vice versa. This methodology ensures a comprehensive, systematic approach to reviewing the literature on diet and PD, addressing the stated objectives and providing a robust evidence base for the identified themes in the results.

Results

The following are the details of the 11 included studies in Table 3. The selected 14 studies scored for quality 3 and above on the basis of MMAT given in Table 4.

**Table 3:** Summary of studies on diet and Parkinson's disease NHANES: The National Health and Nutrition Examination Survey; AHEI: Association between alternative healthy eating index; aMED: Allied and Complementary Medicine Database; PD: Parkinson's disease; MDS-UPDRS: Movement Disorder Society-Unified Parkinson’s Disease Rating Scale; MoCA test: The Montreal Cognitive Assessment;

Sr. no.	Author year	Study type	Population	Methodology	Outcome measures	Key findings
1	Gao et al. (2007) [34]	Prospective cohort	49,692 men and 81,676 women	Principal components analysis, AHEI, and aMED	PD risk	Prudent dietary pattern (high intake of fruits, vegetables, fish) was inversely associated with PD risk
2	Alcalay et al. (2012) [35]	Case-control	257 PD patients, 198 controls	Mediterranean diet adherence score	PD status, age-at-onset	Higher Mediterranean diet adherence associated with reduced PD odds and later age-at-onset
3	Mischley et al. (2017) [36]	Cross-sectional	1,053 PD patients	Food frequency questionnaire	PD progression rate	Fresh vegetables, fruits, nuts, fish, olive oil associated with reduced PD progression; canned foods, soda, fried foods associated with faster progression
4	Phillips et al. (2018) [37]	Randomized controlled trial	47 PD patients	Low-fat vs. ketogenic diet	MDS-UPDRS scores	Both diets improved symptoms; ketogenic diet showed greater improvements in non-motor symptoms
5	Paknahad et al. (2020) [38]	Randomized controlled trial	80 PD patients	Mediterranean diet intervention	Cognitive function (MoCA test)	Mediterranean diet improved executive function, language, attention, and memory in PD patients
6	Rusch et al. (2021) [39]	Single-arm pilot study	PD patients (number not specified)	5-week Mediterranean diet intervention	Constipation, gut microbiota	Mediterranean diet improved constipation and modified gut microbiota in PD patients
7	Kwon et al. (2023) [22]	Case-control	98 PD patients, 83 controls	Healthy Eating Index (HEI)-2015	Diet quality, PD symptoms	PD patients had lower diet quality; poor diet associated with chronic constipation
8	Liu et al. (2023) [40]	Cross-sectional	10,651 adults (NHANES data)	Dietary intake analysis	PD risk	Higher iron intake linked to increased PD risk; higher vitamin K and C intake linked to decreased risk
9	Kwon et al. (2024) [41]	Cross-sectional	85 PD patients	Diet quality assessment, gut microbiome analysis	Gut microbiota composition	Healthy diet and fiber intake associated with anti-inflammatory bacteria; added sugar with pro-inflammatory bacteria
10	Shokri-Mashhadi et al. (2024) [42]	Case-control	105 PD patients, 215 controls	Food frequency questionnaire, principal component analysis	PD risk	Traditional, healthy, and light dietary patterns associated with lower PD risk; Western pattern with higher risk
11	Tidman et al. (2024) [43]	Longitudinal pilot study	7 PD patients	24-week low-carbohydrate high-fat (LCHF) diet intervention	Motor and non-motor symptoms, biomarkers, quality of life	LCHF diet improved biomarkers, cognition, mood, motor and non-motor symptoms, and quality of life in PD patients

The quality of studies is given in Table 4.

**Table 4:** MMAT quality assessment of included studies MMAT: Mixed Method Appraisal Tool

Sr. no.	Author (year)	Study design	MMAT score	Key quality points
1	Gao et al. (2007) [34]	Prospective cohort	4/5	Large sample size, long follow-up, adjusted for confounders
2	Alcalay et al. (2012) [35]	Case-control	3/5	Validated food questionnaire, adjusted for confounders
3	Mischley et al. (2017) [36]	Cross-sectional	3/5	Large sample size, comprehensive dietary assessment
4	Phillips et al. (2018) [37]	Randomized controlled trial	4/5	Randomization, high completion rate, objective outcomes
5	Paknahad et al. (2020) [38]	Randomized controlled trial	4/5	Randomization, validated cognitive assessment tool
6	Rusch et al. (2021) [39]	Single-arm pilot study	3/5	Objective outcomes, microbiota analysis
7	Kwon et al. (2023) [22]	Case-control	4/5	Comprehensive dietary assessment, adjusted for confounders
8	Liu et al. (2023) [40]	Cross-sectional	3/5	Large sample size, propensity score matching
9	Kwon et al. (2024) [41]	Cross-sectional	4/5	Comprehensive microbiome analysis, adjusted for confounders
10	Shokri-Mashhadi et al. (2024) [42]	Case-control	3/5	Newly diagnosed PD cases, validated food questionnaire
11	Tidman et al. (2024) [43]	Longitudinal pilot study	3/5	Mixed-methods approach, comprehensive outcome measures

Synthesis of Results

The studies included in this systematic review collectively provide compelling evidence for the role of diet in PD risk, progression, and symptom management. The research spans various study designs, including prospective cohorts, case-control studies, randomized controlled trials, and cross-sectional analyses, offering a multifaceted view of the diet-PD relationship.

Key Themes

Mediterranean diet and PD risk/progression: Multiple studies consistently demonstrate the benefits of a Mediterranean-style diet in PD. Alcalay et al. found that higher adherence to a Mediterranean diet was associated with reduced odds of PD and later age-at-onset. Paknahad et al. showed improvements in cognitive function among PD patients following a Mediterranean diet intervention. Rusch et al. observed improvements in constipation symptoms and beneficial changes in gut microbiota with Mediterranean diet adherence [35,38,39].

Dietary patterns and PD risk: Several studies identified specific dietary patterns associated with PD risk. Gao et al. found that a prudent dietary pattern rich in fruits, vegetables, and fish was inversely associated with PD risk. Conversely, Shokri-Mashhadi et al. reported that adherence to a Western dietary pattern increased PD risk, while traditional, healthy, and light dietary patterns were associated with lower risk [34,42].

Specific nutrients and PD risk: Liu et al. identified associations between specific nutrients and PD risk in a large cross-sectional study. Higher dietary iron intake was linked to increased PD risk, while higher intakes of vitamins K and C were associated with decreased risk. These findings suggest potential protective effects of certain micronutrients [40].

Diet quality and PD symptoms: Kwon et al. found that PD patients generally had lower diet quality compared to controls, as measured by the Healthy Eating Index (HEI). Poor diet quality was associated with chronic constipation in PD patients, highlighting the potential impact of diet on non-motor symptoms [22].

Ketogenic and low-carbohydrate diets: Two studies explored the effects of ketogenic or low-carbohydrate diets on PD symptoms. Phillips et al. compared low-fat and ketogenic diets, finding that both improved symptoms, with the ketogenic diet showing greater improvements in non-motor symptoms [37]. Tidman et al. reported improvements in biomarkers, cognition, mood, and both motor and non-motor symptoms with a low-carbohydrate, high-fat diet intervention [43].

Diet and gut microbiome in PD: Emerging research suggests that diet influences the gut microbiome composition in PD patients. Kwon et al. found that a healthy diet and higher fiber intake were associated with anti-inflammatory bacteria, while added sugar intake was linked to proinflammatory bacteria. These findings support the potential role of the gut-brain axis in PD pathophysiology [39,41].

Specific foods and PD progression: Mischley et al. (2017) identified specific foods associated with PD progression rates. Fresh vegetables, fruits, nuts, fish, and olive oil were linked to reduced PD progression, while canned foods, soda, and fried foods were associated with faster progression. This granular analysis provides insight into potential dietary recommendations for PD management [36].

Table 5 summarizes the key themes and their relevance with the PD.

**Table 5:** Key themes in diet and Parkinson's disease (PD) research

Key theme	Possible mechanisms	Relevant studies	Additional information
Mediterranean diet benefits	Antioxidant and anti-inflammatory effects. Neuroprotection via polyphenols. Improved gut microbiome composition	Alcalay et al. (2012) [35], Paknahad et al. (2020) [38], Rusch et al. (2021) [39]	Associated with reduced PD risk and later onset. Improves cognitive function in PD patients. Alleviates constipation symptoms
Dietary patterns and PD risk	Cumulative effects of multiple nutrients. Modulation of oxidative stress and inflammation. Influence on insulin sensitivity and metabolic health	Gao et al. (2007) [34], Shokri-Mashhadi et al. (2024) [42]	Prudent/healthy patterns associated with lower risk. Western pattern associated with higher risk. Suggests importance of overall dietary approach rather than single nutrients
Specific nutrients and PD risk	Iron: potential pro-oxidant effects. Vitamins K and C: antioxidant properties. Potential influence on dopamine metabolism	Liu et al. (2023) [40]	High iron intake may increase risk. Vitamins K and C may be protective. Highlights need for balanced micronutrient intake
Diet quality and PD symptoms	Influence on gut-brain axis. Modulation of systemic inflammation. Potential effects on medication absorption	Kwon et al. (2023) [22]	Poor diet quality associated with chronic constipation. Suggests diet as a modifiable factor for non-motor symptoms. May influence overall disease management
Ketogenic and low-carbohydrate Diets	Enhanced mitochondrial function. Neuroprotection via ketone bodies. Potential anti-inflammatory effects	Phillips et al. (2018) [37], Tidman et al. (2024) [43]	May improve both motor and non-motor symptoms. Particularly beneficial for non-motor symptoms. Potential for metabolic health improvements
Diet and gut microbiome in PD	Modulation of gut permeability. Influence on production of neuroactive compounds. Potential effects on α-synuclein aggregation	Rusch et al. (2021) [39], Kwon et al. (2024) [41]	Healthy diet promotes anti-inflammatory bacteria. Added sugars associated with pro-inflammatory bacteria. Supports gut-brain axis involvement in PD
Specific foods and PD progression	Cumulative effects of nutrient profiles. Potential influence on oxidative stress and inflammation. Modulation of gut microbiome	Mischley et al. (2017) [36]	Fresh, whole foods associated with slower progression. Processed and fried foods linked to faster progression. Provides basis for specific dietary recommendations

Discussion

The findings of this systematic review highlight the complex and multifaceted relationship between diet and PD. The synthesis of evidence from the selected studies reveals several key themes that warrant further discussion and comparison with existing literature.

Our review consistently found that adherence to healthy dietary patterns, particularly those rich in fruits, vegetables, whole grains, and fish, was associated with a reduced risk of PD. This aligns with previous reviews, such as the one by Chan et al., which suggested that Mediterranean-style diets may have neuroprotective effects. More aligned with our current review, Rees et al. suggested that a healthy diet consisting of fruits, vegetables, fish, and whole grains can reduce the risk of PD [25,29].

The inverse association between prudent dietary patterns and PD risk observed in our review supports the findings of Gao et al., who reported a 22% lower risk of PD in individuals adhering to a prudent diet. Gao et al.'s study, included in our review, provides strong evidence for the protective effects of a diet rich in fruits, vegetables, and fish [34].

Conversely, our review identified an increased PD risk associated with Western-style dietary patterns, characterized by high intake of red and processed meats, refined grains, and high-fat dairy products. This is consistent with the findings of a large prospective study by Maraki et al., which reported a positive association between a Western diet and PD risk [44]. The mechanisms underlying these associations may involve modulation of oxidative stress, neuroinflammation, and gut microbiome composition, as suggested by Ayten et al. and Bisaglia [45,46].

Our review highlighted the potential neuroprotective effects of certain antioxidants and micronutrients in PD. The study by Liu et al. found that higher iron intake was associated with increased PD risk, while higher vitamin K and C intake was associated with decreased risk [40]. These findings add to the growing body of evidence on the role of specific nutrients in PD pathogenesis and are in line with the review by Zeng et al., which emphasized the potential role of iron accumulation in PD and the protective effects of certain vitamins [47].

However, it's important to note that not all studies have shown consistent results, and the benefits of antioxidant supplementation in PD remain controversial, as highlighted by Fahn [48]. This underscores the need for further research to elucidate the specific roles of individual nutrients in PD risk and progression.

The emerging role of the gut microbiome in PD pathogenesis and progression was a significant theme in our review. The study by Kwon et al. observed associations between diet, gut microbiome composition, and PD, which are consistent with the growing body of literature on the gut-brain axis in PD [41]. Scheperjans et al. previously reported alterations in gut microbiota composition in PD patients, and our findings support the notion that dietary interventions may modulate these alterations [49].

The potential of prebiotic and probiotic interventions in PD management, as suggested by our review, aligns with recent clinical trials. For instance, Tamtaji et al. reported improvements in motor function and oxidative stress markers in PD patients following probiotic supplementation [50]. However, larger and longer-term studies are needed to confirm these effects and elucidate the underlying mechanisms.

Our review found that higher diet quality was associated with reduced severity of certain PD symptoms, particularly constipation. The study by Kwon et al. showed that PD patients generally had lower diet quality compared to controls, and poor diet quality was associated with chronic constipation [22]. This is consistent with the findings of Jackson et al., who reported improvements in gastrointestinal symptoms in PD patients with dietary interventions. The potential impact of diet on non-motor symptoms of PD underscores the importance of considering dietary interventions as part of comprehensive PD management strategies.

The potential benefits of specialized diets, such as the ketogenic diet, in PD management were noted in our review. Phillips et al. and Tidman et al. reported improvements in motor and non-motor symptoms in PD patients following ketogenic or low-carbohydrate diet interventions. This aligns with emerging research on metabolic approaches to neurodegenerative diseases. However, the long-term safety and efficacy of such diets in PD require further investigation [37,43].

Our review highlighted the potential impact of dietary factors on cognitive function in PD, particularly in the study by Paknahad et al., which showed improvements in cognitive function among PD patients following a Mediterranean diet intervention. While specific studies on the Mediterranean-DASH Intervention for Neurodegenerative Delay (MIND) diet in PD are limited, our findings suggest that similar dietary approaches may benefit cognitive function in PD patients [38].

Limitations of the study

Despite the comprehensive nature of this review, several limitations must be acknowledged as the included studies varied widely in design, population characteristics, dietary assessment methods, and outcome measures, making direct comparisons challenging. Moreover, many of the included studies were observational, limiting causal inferences about the relationship between diet and PD. Also, the dietary assessment in most studies relied on self-reported data, which can be subject to recall bias and measurement error. Furthermore, few studies provided long-term follow-up data, limiting our understanding of the long-term effects of dietary interventions in PD. Finally, there was a lack of standardized approaches to dietary assessment and PD outcome measures across studies, potentially affecting the comparability of results.

Future recommendations

Based on the findings and limitations of this review, we propose the following recommendations for future research: To conduct well-designed, long-term randomized controlled trials to establish causal relationships between dietary interventions and PD outcomes, to utilize advanced biomarkers and neuroimaging techniques to provide more objective measures of disease progression and mechanistic insights, to investigate the potential synergistic effects of dietary interventions with conventional PD treatments, and to explore the integration of nutrigenomics and personalized nutrition approaches in PD research, as suggested by Cederholm.

Furthermore, this study recommends to conduct larger studies on the effects of specialized diets (e.g., ketogenic, low carbohydrate) on PD symptoms and progression and to investigate the long-term effects of dietary interventions on PD risk and progression, particularly in high-risk populations. Finally, this study proposes to develop and validate standardized dietary assessment tools specific to PD research and to explore the potential of targeted probiotic and prebiotic interventions in PD management, based on gut microbiome findings.

## Conclusions

This systematic review provides compelling evidence for the significant role of diet in PD risk, progression, and symptom management. The findings suggest that adherence to healthy dietary patterns, particularly those rich in fruits, vegetables, whole grains, and fish, may reduce PD risk, while Western-style diets high in processed foods and red meat may increase risk. Specific nutrients, especially antioxidants, and certain vitamins show potential neuroprotective effects. The review highlights the emerging importance of the gut-brain axis in PD, suggesting that dietary interventions targeting gut health could be beneficial. Additionally, diet quality appears to influence PD symptoms, particularly non-motor symptoms like constipation. While specialized diets such as the ketogenic diet show promise in managing PD symptoms, further research is needed to establish their long-term efficacy and safety. The complex nature of the diet-PD relationship necessitates further research to develop evidence-based, personalized dietary recommendations for PD prevention and management. As our understanding of the interplay between diet, genetics, and environmental factors in PD continues to evolve, the potential for tailored nutritional strategies in PD care becomes increasingly apparent.

In conclusion, dietary interventions have the potential to serve as a complementary approach to existing PD therapies. However, larger, longer-term studies are needed to confirm these effects and elucidate the underlying mechanisms linking diet and PD. The development of evidence-based dietary guidelines for PD prevention and management should be a priority for future research in this field.
